# Editorial: Traditional medicine and phytochemicals for neurodegenerative diseases treatment: application of interdisciplinary technologies in novel therapeutic target and drug discovery

**DOI:** 10.3389/fnins.2023.1268710

**Published:** 2023-08-08

**Authors:** Chuipu Cai, Jiansong Fang, Hanzhong Ke

**Affiliations:** ^1^Division of Data Intelligence, Department of Computer Science, Key Laboratory of Intelligent Manufacturing Technology of Ministry of Education, College of Engineering, Shantou University, Shantou, China; ^2^Science and Technology Innovation Center, Guangzhou University of Chinese Medicine, Guangzhou, China; ^3^Department of Cancer Immunology and Virology, Dana-Farber Cancer Institute, Harvard Medical School, Boston, MA, United States

**Keywords:** neurodegenerative disease, traditional medicine, phytochemicals, drug discovery, Alzheimer's disease, Parkinson's disease, bioinformatics

Neurodegenerative diseases are a group of disorders characterized by the progressive degeneration and dysfunction of the nervous system, such as Alzheimer's disease (AD) and Parkinson's disease (PD). These diseases primarily affect neurons, which are the building blocks of the central nervous system (brain and spinal cord), leading to a gradual decline in cognitive function, movement control, and overall neurological function. In the past decades, the prevalence of neurodegenerative disorders is on the rise, partially attributable to extensions in lifespan (Heemels, 2016). According to the latest report by the World Health Organization (15 March 2023), the global prevalence of dementia stands at over 55 million individuals, with a yearly increase of nearly 10 million cases. The rapidly increasing prevalence of neurodegenerative diseases is bringing significant public health problems. Traditional medicine and phytochemicals, which typically contain abundant chemical scaffolds and multiple targets, are proving to be an invaluable chemical library for novel drug development against neurodegenerative diseases. Previous clinical evidence and pharmacological studies have demonstrated that traditional medical practice and plant-derived natural products have the capacity to alleviate cognitive impairment, motor dysfunction, emotional instability, etc. Unfortunately, the intricate ingredients, indistinct molecular mechanisms and unclear clinical benefit severely hinder the advance (Wu et al., 2020). In recent years, interdisciplinary collaboration strategy, especially the comprehensive application of both *in silico* approaches and wet-lab assays, has achieved remarkable success in speeding up drug discovery and mechanism elucidation (Li et al., 2021). The Research Topic compiles a series of articles utilizing interdisciplinary technologies, including deep learning algorithm, network pharmacology, molecular docking, untargeted metabolomics analysis, high-throughput mRNA-sequencing analysis, as well as systematic review and meta-analysis, providing new insights into the novel phytochemicals, therapeutic targets, and molecular mechanisms for traditional medicine against neurodegenerative diseases.

Traditional Chinese medicine (TCM) has extensively employed herbal remedies for treating neurodegenerative diseases for a long history. In recent years, the utilization of complementary therapies has surged, particularly in Western societies, leading to the growing integration and modernization of TCM in contemporary medical practices (Xu et al., 2013). In this Research Topic, Yagüe et al. provided an insightful perspective on the achievements and challenges of TCM, along with highlighting how the advancement of novel technologies facilitates the integration of TCM into Western medical practice. They summarized the main bottlenecks in the modernization of arcane Chinese herbal medicine: lack of standardization, safety concerns and poor quality of clinical trials, as well as the ways these are being overcome. It is beneficial to put effort into these aspects, but the holistic nature of TCM requires a paradigm shift such that novel approaches, tools, and methodologies can be developed. Through a shift in perspective, transitioning from a “one-target, one-drug” approach to a “network-target, multiple-component therapeutics” paradigm, network pharmacology, in conjunction with other systems biology, omics methodologies and TCM databases, will pave the way toward TCM modernization.

The preparation in TCM refers to the combination of herbs used for treatment. In this Research Topic, two Chinese herbal preparations, Gancao nourishing *yin* decoction (GCNY) and Bushen Tiansui formula (BSTSF), have been investigated for their neuroprotective mechanisms. Chen et al. tried to explore the alleviate impact of the GCNY formula on the peroxidative stress levels in H_2_O_2_-modeled human fetal brain-derived primary microglia cells. High-throughput mRNA-sequencing analysis were conducted to identify the differential expressed genes and further figure out how GCNY-medicated serum acts on HMC-3 cells based on the overall genotype changes. Li et al. evaluated the effect of BSTSF treatment on cognitive dysfunction and investigated its neuroprotective mechanisms against AD by utilizing LC-MS/MS-based untargeted metabolomics analysis in the cerebral cortex and hippocampus of AD rats. They showcased that BSTSF exhibited significant alleviation in memory deficits and mitigated the characteristic histopathological changes in AD rats. The therapeutic effects of BSTSF on AD were found to be associated with the regulation of cysteine and methionine metabolism, d-glutamine and d-glutamate metabolism, as well as phospholipid metabolism. However, further in-depth researches are necessary to uncover the complicated interactions between the mixture system of ingredients and molecular mechanisms in the future.

The therapeutic potential of phytochemicals or herbal extracts like Ginkgo biloba extract (GBE) toward neurodegenerative diseases have garnered increasing attention. Numerous studies have demonstrated the neuroprotective effects of a mixture of GBE in various PD animal and cell culture models. However, the inherent variability, mixture nature and multi-target effects of GBE hinder the investigation of underlying molecular mechanisms. To overcome this obstacle, it is crucial to elucidate the specific effects of individual active components within GBE through effective approaches. Yan et al. established a cell-type-specific targets and compound network through the integration of data from single-nuclei RNA sequencing and existing drug datasets. By employing a deep learning algorithm and molecular docking, they tended to identify drug-target interactions between 25 active compounds in GBE and 47 PD targets in three cell types. Results suggested relatively strong interactions between ginkgolides and several PD targets participating in core biological pathways. This knowledge may help to the understanding of GBE mechanisms in treating PD, but further validation by benchwork experiments is necessary to confirm the actual interactions between the predicted drug-target interactions (DTI) identified in this study.

Treatment based on syndrome differentiation (SD) is the basic principle of TCM that based on a holistic theory and personalized treatment according to the time-order progression of diseases. Jun et al. performed a systematic review and meta-analysis of 13 randomized placebo-controlled clinical trials involving 843 participants, aiming to evaluate the efficacy of herbal medicine prescribed based on SD for PD treatment. The findings suggested that combining Western medicine and herbal medicine based on SD diagnosis, offers additional benefits in the treatment of PD, but further studies with rigorous designs and longer follow-up periods are still needed. Park et al. systematically summarized the possible interdisciplinary biomarkers including transcriptomic and neuroimaging studies by SD-based herbal medicine therapy for AD. Their work aimed to explore potential peripheral blood biomarkers of multi-target and multi-time herbal medicine therapy toward AD, which expected to be reflected in different disease stage progressions for patients with cognitive impairment.

Neurodegenerative diseases exhibit complex pathophysiology, involving neuroinflammation and neuronal death as significant contributors. Pyroptosis (“fiery death”), a form of inflammatory cell death, mediated by a family of pore-forming proteins called Gasdermins, has been considered as a downstream process triggered by inflammasome activation (Moujalled et al., 2021). Consequently, investigating pyroptosis has become an attractive area of study in neurodegenerative diseases research. Previous studies have validated the efficacy of TCM formulas, extracts, and acupuncture in ameliorating neurodegenerative symptoms through the suppression of pyroptosis. In this Research Topic, Liao et al. provided an overview of the molecular mechanisms driving pyroptosis, the connections between pyroptosis and neurodegenerative diseases, and potential therapeutic approaches within formulas and extracts to inhibit pyroptosis for the treatment of neurodegenerative diseases.

In summary, the current article Research Topic “*Traditional Medicine and Phytochemicals for Neurodegenerative Diseases Treatment: Application of Interdisciplinary Technologies in Novel Therapeutic Target and Drug Discovery*” showcased the new perspectives and applications of interdisciplinary strategy in the investigation of traditional medicine and phytochemicals against neurodegenerative diseases. We are delighted to see that the convergence of pharmacology and advanced technologies in related subfields, such as omics, artificial intelligence, bioinformatics, etc., has brought hope for addressing issues at the bridge between traditional medicine/phytochemicals and neurodegenerative disease therapies. However, it should be noted that basic research in this area is still challenging, and there is still a long journey ahead before achieving successful clinical translation.

## Author contributions

CC: Writing—original draft, Writing—review and editing. JF: Writing—review and editing. HK: Writing—review and editing.

## References

[B1] Heemels M. T. (2016). Neurodegenerative diseases. Nature 539, 179. 10.1038/539179a 27830810

[B2] Li D. Cai C. Liao Y. Wu Q. Ke H. Guo P.. (2021). Systems pharmacology approach uncovers the therapeutic mechanism of medicarpin against scopolamine-induced memory loss. Phytomedicine 91, 153662. 10.1016/j.phymed.2021.153662 34333326

[B3] Moujalled D. Strasser A. Liddell J. R. (2021). Molecular mechanisms of cell death in neurological diseases. Cell Death Differ. 28, 2029–2044. 10.1038/s41418-021-00814-y 34099897 PMC8257776

[B4] Wu Q. Chen Y. Gu Y. Fang S. Li W. Wang Q.. (2020). Systems pharmacology-based approach to investigate the mechanisms of Danggui-Shaoyao-san prescription for treatment of Alzheimer's disease. BMC Complement. Med. Ther. 20, 282. 10.1186/s12906-020-03066-4 32948180 PMC7501700

[B5] Xu Q. Bauer R. Hendry B. M. Fan T. P. Zhao Z. Duez P.. (2013). The quest for modernisation of traditional Chinese medicine. BMC Complement Altern. Med. 13, 132. 10.1186/1472-6882-13-132 23763836 PMC3689083

